# Computational prediction of lncRNA-mRNA interactionsby integrating tissue specificity in human transcriptome

**DOI:** 10.1186/s13062-017-0183-4

**Published:** 2017-06-08

**Authors:** Junichi Iwakiri, Goro Terai, Michiaki Hamada

**Affiliations:** 1 Graduate School of Frontier Sciences, University of Tokyo, 5–1–5 Kashiwanoha, Kashiwa, Chiba, 277–8562 Japan; 2 INTEC Inc, 1–1–25 Shin-urashima-cho, Kanagawa-ku, Yokohama, Kanagawa, 221–8520 Japan; 3 Department of Electrical Engineering and Bioscience, Faculty of Science and Engineering, Waseda University, 55N–06–10, 3–4–1, Okubo Shinjuku-ku Tokyo, 169–8555 Japan; 4 Artificial Intelligence Research Center, National Institute of Advanced Industrial Science and Technology (AIST), 2–41–6 Aomi, Koto-ku, Tokyo, 135–0064 Japan; 5 AIST-Waseda University Computational Bio Big-Data Open Innovation Laboratory (CBBD-OIL), 3–4–1, Okubo Shinjuku-ku, Tokyo, 169–8555 Japan; 6 Institute for Medical-oriented Structural Biology, Waseda University, 2–2, Wakamatsu-cho Shinjuku-ku, Tokyo, 162–8480 Japan; 7 Graduate School of Medicine, Nippon Medical School, 1–1–5, Sendagi, Bunkyo-ku, Tokyo, 113–8602 Japan

**Keywords:** RNA-RNA interaction, Long non-coding RNA, Tissue specificity, RNA-seq, Computational prediction

## Abstract

Long noncoding RNAs (lncRNAs) play a key role in normal tissue differentiation and cancer development through their tissue-specific expression in the human transcriptome. Recent investigations of macromolecular interactions have shown that tissue-specific lncRNAs form base-pairing interactions with various mRNAs associated with tissue-differentiation, suggesting that tissue specificity is an important factor controlling human lncRNA-mRNA interactions.

Here, we report investigations of the tissue specificities of lncRNAs and mRNAs by using RNA-seq data across various human tissues as well as computational predictions of tissue-specific lncRNA-mRNA interactions inferred by integrating the tissue specificity of lncRNAs and mRNAs into our comprehensive prediction of human lncRNA-RNA interactions. Our predicted lncRNA-mRNA interactions were evaluated by comparisons with experimentally validated lncRNA-mRNA interactions (between the TINCR lncRNA and mRNAs), showing the improvement of prediction accuracy over previous prediction methods that did not account for tissue specificities of lncRNAs and mRNAs. In addition, our predictions suggest that the potential functions of TINCR lncRNA not only for epidermal differentiation but also for esophageal development through lncRNA-mRNA interactions.

**Reviewers:** This article was reviewed by Dr. Weixiong Zhang and Dr. Bojan Zagrovic.

**Electronic supplementary material:**

The online version of this article (doi:10.1186/s13062-017-0183-4) contains supplementary material, which is available to authorized users.

## Findings

Recent RNA sequencing (RNA-seq) studies have identified many long noncoding RNAs (lncRNAs) that are expressed across various healthy and cancerous human tissues [1, 2]. According to the statistical analysis conducted as part of the GENCODE Project [3, 4], the number of annotated human lncRNA genes is increasing, whereas the number of annotated small non-coding RNA (small ncRNA) genes and protein-coding genes has been stable in recent years; the number of annotated lncRNA genes has increased from 6496 (version 3c released in 2009) to 15,877 (version 21 released in 2014), whereas the numbers of annotated small ncRNA and protein-coding genes have been stable at around 9000 and 20,000, respectively.

The functions of most of these lncRNAs are still unclear, except for a few well-studied lncRNAs, such as NEAT1 [5] and MALAT1 [6], which are ubiquitously expressed across various human tissues at relatively high levels that are comparable to those of protein-coding transcripts [7]. However, the analysis of a large collection of RNA-seq reads derived from 24 human tissues revealed that the expression levels of most lncRNAs are biased towards occurring in one or a few tissues [8]. Furthermore, the recent integrative analysis of over 7000 RNA-seq samples derived from normal and tumor tissues as well as various cell lines revealed the landscape of expression patterns of lncRNAs in human transcriptome data and highlighted the large number of lncRNA genes (approx. 8000 genes) that are estimated to be specifically expressed in a few normal or cancerous tissues [2]. These studies imply an important function of lncRNAs is the regulation of cell lineage differentiation or cancer development through their tissue-specific expression patterns in the human transcriptome.

Recently, terminal differentiation-induced ncRNA (TINCR), a 3733-nt lncRNA, was also identified to be specifically expressed at a late stage of human epidermal differentiation and to regulate expression levels of various mRNAs by a post-transcriptional mechanism [9]. To analyze the mRNA regulatory mechanism of TINCR in human epidermis, Kretz et al. developed a RNA interactome analysis involving deep sequencing (RIA-Seq) based on a TINCR-specific antisense probe to identify the interacting mRNA targets of TINCR; they reported that TINCR interacted with various mRNAs that regulate epidermal differentiation through TINCR-mRNA base-pairing interactions. Their study suggests the potential function of the various lncRNAs expressed in the specific tissues is to interact with other RNAs via RNA-RNA interactions. Therefore, computational predictions of lncRNA-mRNA interactions would be useful for for more accurately inferring the functions of the tissue-specific lncRNAs in the human transcriptome.

Previously, we developed a computational pipeline for comprehensive predictions of lncRNA-RNA interactions from the primary sequences of lncRNAs and mRNAs in the human transcriptome and provided the predicted lncRNA-RNA interactions [10]. Considering the tissue-specific expression of various lncRNAs as mentioned above, further investigations into the prediction of lncRNA-mRNA interactions is necessary for improved accuracy.

In this study, we investigated the tissue specificity of lncRNA and protein-coding gene expression using RNA-seq data derived from 16-32 different human tissues, and we observed a large fraction of the lncRNAs that exhibited more tissue-specific expression relative to protein-coding genes. Furthermore, we used tissue-specific lncRNA and mRNAs to predict lncRNA-mRNA interactions with improved accuracy by assuming that lncRNAs and their interacting target mRNAs exhibit high expression specificity within the same tissues via the regulatory mechanism of lncRNAs upregulating the interacting target mRNAs through base-pairing interactions. This proposed approach was evaluated using the experimentally-validated TINCR-mRNA interactions, and it exhibited better prediction accuracy than that achieved using only RNA sequences. Our integration of the tissue specificity of human lncRNA and mRNAs into the prediction of lncRNA-mRNA interactions can provide fundamental insights through functional studies of lncRNAs in human tissues.

## Investigation of tissue specificity of human lncRNA and protein-coding genes

**Fig. 1:**
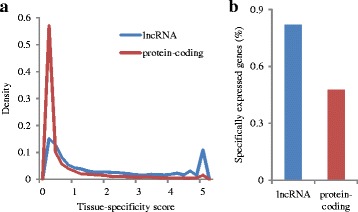
The tissue specificity of human lncRNAs and protein-coding genes. The tissue specificity of 6414 lncRNAs and 17,806 protein- coding genes analyzed using RNA-seq data from the Human Protein Atlas Project [13] (Expression Atlas ID: E-MTAB-2836). **a** Distributions of tissue-specificity scores [11] calculated for lncRNA and protein- coding genes. **b** Fraction of specifically expressed genes determined to be outliers by ROKU [12]

The tissue specificities of human lncRNA and protein-coding genes were investigated by calculating entropy-based tissue-specificity scores from RNA-seq data [11]. The distributions of tissue-specificity scores for 6414 lncRNA and 17,804 protein-coding genes based on the Human Protein Atlas Project RNA-seq data are shown in Fig. 1 a. The expression levels of lncRNAs are biased toward high tissue specificity in comparison with those of protein-coding genes. In particular, there are two peaks in the distribution of lncRNA tissue-specificity scores. The first peak is observed at tissue specificity scores almost equal to zero, which indicates that the corresponding lncRNAs and protein-coding genes are widely expressed across all tissues. The second peak is observed for only lncRNAs at the highest tissue-specificity scores, which indicates that the corresponding lncRNAs are exclusively expressed in single tissues. In addition, we considered the lncRNAs or protein-coding genes with outlier expression levels in at least one tissue as detected by ROKU [12] as *specifically expressed genes*. Most of the lncRNA genes (81.8%) exhibited tissue-specific expression, whereas a smaller fraction of the protein-coding genes (47.7%) exhibited such specificity (Fig. 1 b), P ≤10^−16^, Fisher’s exact test). In addition, similar results shown in Additional file 1 were obtained by analyzing the two RNA-seq datasets derived from the GTEx Consortium and Illumina Body Map Project, respectively. Cabili et al. also reported a similarly strong tissue-specific tendency of lncRNA expression profiles in RNA-seq data from 24 tissues [8]. Our analysis confirmed their results in the different RNA-seq datasets including larger scale RNA-seq studies.

## Collecting lncRNA and protein-coding genes with the same tissue specificity

Recent studies show that tissue-specific lncRNAs upregulate expression levels of various mRNAs via base-pairing interactions between these RNAs [9]. Under this regulatory mechanism, the lncRNAs and their cognate mRNAs would be specifically expressed in the same individual tissues, which suggests lncRNA and mRNA pairs expressed in the same tissues are better candidates for predicting lncRNA-mRNA interactions.

To identify the tissue-specific lncRNA and mRNA pairs from RNA-seq data, the tissues in which each lncRNA or protein-coding gene was specifically expressed were determined to exhibit outlier expression levels using ROKU [12]. For each tissue, the number of the detected lncRNA and protein-coding genes are summarized in Additional files 2, 3, 4 and 5. From the Human Protein Atlas Project RNA-seq data, the largest number of protein-coding genes were reported as testis-specific genes [13]. In addition to confirming this result, we find that a larger fraction of lncRNA genes (40.5%) were also found to be testis-specific relative to protein-coding genes (17.2%).

## Tissue specificity improves prediction of lncRNA-mRNA interactions

We previously developed a computational pipeline for comprehensive prediction of lncRNA-mRNA interactions in human transcriptome data [10]. In this study, the tissue specificities of lncRNAs and mRNAs derived from the expression profiles of several RNA-seq studies were combined with our sequence-based predictions of lncRNA-mRNA interactions to achieve more reliable predictions. This captures the regulatory mechanism of tissue-specific lncRNAs upregulating their interacting target mRNAs, as was observed in TINCR-mRNA interactions. Assuming this mechanism, the lncRNAs and their target mRNAs would be specifically expressed in the same individual tissues, and the collections of these lncRNAs and mRNAs could thus be considered better candidates for predicting lncRNA-mRNA interactions within specific tissues.

**Fig. 2:**
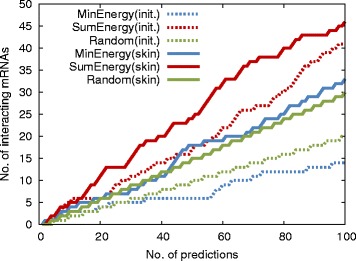
Our predictions of TINCR-mRNA interactions using skin-specific mRNAs. The skin-specific candidate mRNAs were identified from RNA-seq data derived from the Human Protein Atlas Project (Expression Atlas ID:E-MTAB-2836). A combination of two prediction (ranking) methods (MinEnergy and SumEnergy) and two candidate mRNA sets (initial and tissue specific) were used for the predictions. Experimentally validated TINCR-mRNA interactions [9] (considered as true positives) were used for evaluating the prediction results. The *horizontal* axis indicates the number of predicted TINCR-mRNA interactions. The *vertical* axis indicates the number of experimentally validated interactions (i.e., true positives)

To evaluate this approach, experimentally-validated TINCR-mRNA interactions that were observed in skin tissue [9], were used as a case study. From the Human Protein Atlas RNA-seq dataset, TINCR was found to be a skin-, placenta-, and esophagus-specific lncRNA in the outlier analysis (Additional file 6). For the candidate mRNAs in TINCR-mRNA interactions, 5090 experimentally investigated mRNAs with expression levels ≥1 FPKM in at least one tissue were used for the prediction. These mRNAs were considered the *initial candidates* for predicting TINCR-mRNA interactions and were included in our previous predictions of lncRNA-mRNA interactions. Among these initial candidate mRNAs, 285 mRNAs were detected as skin-specific RNAs from the RNA-seq data (i.e., *skin-specific candidates*). In the skin-specific candidate, TINCR-interacting mRNAs were significantly enriched compared with initial candidates (Additional file 6). This suggests that skin-specific mRNAs could be better candidates for predicting TINCR-mRNA interactions. In the prediction of TINCR-interacting mRNAs using the two types of candidate mRNAs, candidate mRNAs were ranked according to two different interacting energies (MinEnergy and SumEnergy, see Materials and methods for details). The prediction performances of TINCR-mRNA interactions using two types of candidate mRNAs are shown in Fig. 2. By using the skin-specific candidates instead of the initial candidates, the number of the true positive mRNAs predicted by SumEnergy ranking was increased (Fig. 2). When using MinEnergy ranking with the skin-specific candidates, the number of true positives also increased compared with that obtained using the initial candidates. These results show that our approach using skin-specific candidate mRNAs provides more reliable predictions of TINCR-mRNA interactions. Note that the overall prediction performance of SumEnergy ranking exceeded that of MinEnergy ranking as reported previously, as there are several local interacting sites that were observed among the TINCR-mRNA interactions [9].

The other 31 tissue-specific candidate mRNAs (shown in Additional file 2) were also used for predicting TINCR-mRNA interactions (shown in Additional files 6 and 7). Interestingly, the number of true positive mRNAs was increased only when esophagus-specific candidates were used for the prediction of the interactions. This improvement was comparable to the results of skin-specific candidates. This result is caused by the expression of TINCR in these two tissues. Thus, it is possible that the TINCR-mRNA interactions upregulating the expression of various mRNAs are not only important for epidermal differentiation but also for esophageal development. Similar results were observed by using a different RNA-seq dataset produced by the GTEx Consortium, which included 30 tissues (shown in Additional files 8 and 9). In addition, using vagina-specific candidates also improved the prediction results in this RNA-seq dataset.

## Conclusion

In this study, we have integrated RNA-seq datasets derived from various human tissues into the predictions of lncRNA-mRNA interactions by using the tissue specificity of human transcripts. To achieve more reliable predictions of the lncRNA-mRNA interactions occurring through the regulatory mechanism of tissue-specific lncRNAs upregulating the expression level of their interacting target mRNAs, the lncRNAs and mRNAs sharing the same tissue specificities were selected as candidates for the subsequent prediction of lncRNA-mRNA interactions. This approach was evaluated using experimentally validated TINCR-mRNA interactions, and we improved the prediction accuracy for these interactions by using tissue-specific mRNAs as candidates. Through the predictions of TINCR-mRNA interactions as shown in this study, we showed the potential for integrating various RNA-seq data into comprehensive predictions of lncRNA-mRNA interactions based on the tissue specificities of lncRNAs and mRNAs estimated from RNA-seq data that include multiple tissue samples. This approach is applicable to potential lncRNAs that regulate the expression levels of various mRNAs through lncRNA-mRNA base-paring interactions because a large fraction of lncRNAs are expressed in a tissue-specific manner in the human transcriptome.

## Materials and methods

### RNA-seq data from various human tissues

In this study, four different RNA-seq datasets, which included more than 10 human tissues, were used to investigate the tissue specificity of human RNAs (shown in Additional file 10). The first RNA-seq dataset was produced by the Human Body Map (Illumina Body Map) Project, which included RNA samples derived from 16 tissues, and was previously used to analyze the tissue specificity of various types of human transcripts [8]. The second RNA-seq dataset, derived from 32 tissues isolated from 122 human individuals, was recently released by the Human Protein Atlas project [13]. The third RNA-seq dataset was derived from 30 representative tissues by the GTEx Consortium [14]. The last RNA-seq dataset was produced by Epigenome Roadmap Project from 19 human tissues [15]. Note that the last RNA-seq dataset was derived from individuals at a developmental stage (fetuses with congenital defects) different from that of the first three RNA-seq datasets (i.e., adult humans). Expression levels (given as fragments per kilobase of exon per million mapped fragments, FPKM) of all the human genes were obtained from the Expression Atlas on 10 March 2016 [16]. Definitions of lncRNA and protein-coding genes were imported from the GENCODE annotation (Release 19) [3].

### Analysis of tissue specificity from RNA-seq data

Every gene expression profile derived from RNA-seq data was investigated using the following two methods. In the first method, a tissue-specificity score [11] was used to investigate tissue specificity of the expression pattern of each lncRNA and protein-coding gene. This score, ranging from 0 (ubiquitously expressed) to a maximum value (specifically expressed only in one tissue), is based on Shannon entropy, which has been frequently used to measure tissue specificity from expression profile data derived from microarray and RNA-seq data [8, 11, 12, 17]. Shannon entropy was calculated using ROKU [12], and converted to a tissue-specificity score. In the second method, an outlier analysis was applied to each gene expression profile to detect the tissues exhibiting highly outlying expression levels. The genes with outlier expressions in at least one tissue were considered *specifically expressed genes*. ROKU was also used for this outlier analysis.

For the ROKU input data, all expression levels estimated in FPKM were log2-transformed (after adding 1 to raw values in order to avoid infinite values). The lncRNA and protein-coding genes with expression levels ≥1 FPKM in at least one tissue were used for our analysis. ROKU was implemented as a function of the TCC library in Bioconductor [18].

### Comprehensive prediction of lncRNA-mRNA interactions in human transcriptome data

We previously developed a series of computational pipelines including various computational tools for sequence analysis, such as Raccess [19], TanTan [20], LAST [21, 22], IntaRNA [23], and RactIP [24], for predicting human lncRNA-RNA interactions [10]. Furthermore, all predicted human lncRNA-RNA interactions are available from our database (http://rtools.cbrc.jp/cgi-bin/RNARNA/index.pl). Our database contains the lncRNA-mRNA and lncRNA-lncRNA interactions between 23,898 lncRNAs and 20,185 mRNA sequences obtained from the GENCODE Project (http://www.gencodegenes.org/releases/19.html). In this database, each possible pair of RNAs are ranked according to two interaction energies (MinEnergy and SumEnergy) calculated from the local interaction energies between the two RNA sequences. In the SumEnergy calculation, a -16 kcal/mol energy cutoff was applied to obtain better predictions. (see our previous study, [10], for further details). The predicted lncRNA-mRNA interactions including these two types of interacting energies were used for the analysis.

## Reviewers’ comments

### Reviewer’s report 1

Weixiong Zhang, Washington University

The current study extended an early work by the authors to include tissue specificity to RNA-RNA interaction analysis so as to increase accuracy for predicting possible interactions between long noncoding RNAs (lncRNAs) and mRNAs in human. The authors examined a large number of RNA-seq datasets from many human tissues in the public domain to compare tissue-specificity scores for a list of lncRNAs and a list of mRNAs and identify the tissues where these lncRNAs and mRNAs are most likely to express. Analysis of RNA-RNA interaction was then performed on pairs of lncRNAs and mRNAs. They then further analyzed the accuracy of the prediction using the experimental data on a particular lncRNA TINCA as a validation to show the improved prediction accuracy after using tissue specificity. While the work is interesting, it is incremental. No new algorithm or method for predicting RNA-RNA interaction was developed. The current study followed closely the work in an early paper in BMC Genomics. Adopting tissue specificity is useful, but it’s also straightforward. The paper was well written and easy to read.

The main idea is simple and the work is incremental. To maintain the quality of the journal, I won’t recommend to publish this paper.

Authors’ Response: *In this study, we highlight following points that were not included in the previous paper.*

- *Analysis of expression patterns shows that most of human lncRNAs are highly specific to particular tissues (Fig.* *1* *and Additional file* *1* *)*.
- *The tissue-specific lncRNAs and their interacting partner mRNAs exhibit similar tissue-specificity, which could be beneficial for predicting tissue-specific lncRNA-mRNA interactions (Fig.* *2* *)*.
- *Our predictions suggest that TINCR-mRNA interactions, originally identified in skin tissues, could also be important for esophagus development (Additional files* *7* *and* *9* *)*.

*We submitted this manuscript as Discovery Notes which is intended for the brief reports of specific discoveries, since our approach would be useful, whereas it is conventional and straightforward as pointed out by the reviewer*.

### Reviewer’s report 2

Bojan Zagrovic, Mediterranean Institute for Life Sciences

The authors present details of an application of a previously published computational paradigm for predicting lncRNAs-mRNA interactions, but now with an inclusion of tissue specificity. By focusing on a particular experimentally well characterize lncRNA (TINCR) they attempt to show that integrating tissue specificity leads to better predictions. 1. My main point of criticism is the fact that the analysis to support the authors’ claim is extremely basic and rudimentary. In short, they just show that the inclusion of tissue specificity increases the fraction of true positives in the top 100 predictions. However, they do not provide a detailed analysis of ROC curves which would include false positive rates, or similar analyses. Also, their principal result could simply be a consequence of the fact the in some tissues e.g. skin and esophagus, the fraction of true binders is greater than elsewhere. In this sense, any prediction algorithm (including even guessing at random) would show similar features when it comes to an increase in true positives upon inclusion of tissues specificity data. A more detailed statistical analysis is required in any case to clearly differentiate predictions without and with tissue specificity included.

Authors’ Response: *We are grateful to the reviewer for the helpful suggestions. As mentioned by the reviewer, the inclusion of tissue-specificity data could increase the fraction of true interacting mRNAs within the dataset (subset of initial dataset), which improves the number of true positives in the top 100 predictions regardless of the prediction algorithms (including SumEnergy, MinEnergy or random guessing). In order to clarify the improvements of our prediction over the random guessing, we added the random guess into Fig.* *2* *, Additional files* *7* *and* *9* *in the revised manuscript. These figures show that SumEnergy predictions provided more true positives than the random guess, even though tissue-specific datasets were used for prediction.*

*In addition, we have performed the statistical analysis of the enrichment of true interacting RNAs within the tissue-specific dataset compared with initial dataset (Additional files* *6* *and* *8* *). The analysis clearly shows significant enrichments of true interacting RNAs in skin (reported in Kretz et al. Nature, 2013), esophagus (not reported previously), and a few tissues. TINCR was also detected to be specifically expressed in the most of these tissues*.

*It is noted that the analysis of ROC curves involving true/false positive rates is not appropriate for comparison of the predictions using different datasets (initial and tissue-specific datasets), because the number of interacting (positive), non-interacting (negative) RNAs, and ratio of positive/negative samples within the tissue-specific datasets (e.g. skin and esophagus) are different from initial dataset (shown as Additional files* *6* *and* *8* *in the revised manuscript)*.

2. Concerning the comment in 1, by focusing on a particular tissue in which the targets of a given molecule are known to reside, one immediately improves the odds of being successful. The authors should address this challenge by providing a more detailed analysis of the predictions in the cases of tissue which are not know to harbor any partners.

Authors’ Response: *We thank the reviewer for mentioning the important issue. To our knowledge, TINCR-mRNA interactions was originally identified in skin tissues, and not known in esophagus previously. However, the additional statistical analysis (as suggested by the reviewer in comment1) shows a significant enrichment of interacting mRNAs in esophagus (Additional files* *6* *and* *8* *). Furthermore, using the esophagus-specific candidates for predicting TINCR-mRNA interactions could increase true positive predictions as observed by using skin-specific candidates (Additional files* *7* *and* *9* *).*

*Currently, our aim of this study is to show the effective exploitation of the tissue-specificity for improving sequence-based prediction of lncRNA-mRNA interactions. Further analysis and predictions for the other lncRNAs and their partners is beyond the scope of the Discovery Notes which is intended for the brief reports of specific discoveries. We mentioned this important issue at the end of* *Conclusion* *section*.

3. For a properly thorough analysis of their algorithm and the benefits of including tissue specificity, the authors should look into a much larger number of experimentally well-characterized systems. As is, the authors’ conclusions are in danger of being seen as only anecdotal.

Authors’ Response: *We agree with the reviewer’s comment regarding the larger number of experimentally identified lncRNA-mRNA interactions for the more rigorous evaluation of our predictions. We are always looking for novel experimentally identified lncRNA-mRNA interactions. Currently, however, the number of experimentally well-characterized lncRNA-mRNA interactions is quite limited, because experimental identifications of lncRNA-mRNA interactions are still difficult. Recently, three experimental methods (PARIS* ^1^ *, LIGR-seq* ^2^ *and SPLASH* ^3^ *) were developed for a comprehensive identification of human RNA-RNA interactions. These methods could identify the interactions between mRNAs and well-known ncRNAs (such as rRNAs, snoRNAs) which are highly and ubiquitously expressed in many tissues. However, only a few lncRNA-mRNA interactions were identified by these methods due to the low-expression levels of various lncRNAs and their tissue-specificities.*

1. *Lu, Zhipeng, et al., Cell 165.5 (2016): 1267-1279*.
2. *Sharma, Eesha, et al., Molecular cell 62.4 (2016): 618-626*.
3. *Aw, Jong Ghut Ashley, et al., Molecular cell 62.4 (2016): 603-617*.

The Supp. Mat. Figures 1-3 should be consolidated into one figure, for the ease of reading.

Authors’ Response: *In accordance with the reviewer’s suggestion, we concatenated the three figures into one figure in the revised manuscript (new figure: Additional file* *1* *).*

In multiple places the authors refer to the K computer, used in the study, as one of the fastest super-computers in the world. This should be removed as it does not provide any particularly useful information to the reader, especially in the absence of significant benchmarks or hardware/accessibility details. It is likely that the authors did not have the entire K computer to their disposal, in which sense these other details become critical.

Authors’ Response: *In the revised manuscript, we removed the useless sentences as suggested by the reviewer.*

It should be Fisher’s exact test.

Authors’ Response: *We thank the reviewer for pointing it out. We corrected this typographical error in the revised manuscript.*

## Additional files

Additional file 1 (a) Tissue specificity of 6852 lncRNAs and 17,612 protein-coding genes analyzed using human RNA-seq data from the GTEx Consortium (Expression Atlas ID: E-MTAB-2919). (b) Tissue specificity of 5105 lncRNAs and 17,017 protein-coding genes analyzed using human RNA-seq data from the Human Body Map Project (Expression Atlas ID: E-MTAB-513). (c) Tissue specificity of 4973 lncRNAs and 16,164 protein-coding genes analyzed using human RNA-seq data from the NIH Epigenomics Roadmap project (Expression Atlas ID: E-MTAB-3871). (left) Distributions of tissue-specificity scores [11] calculated for lncRNA and protein-coding genes. (right) Fraction of specifically expressed genes in one or more tissues that were determined to be outliers by ROKU [12]. (PDF 15 kb)

Additional file 2 Number of tissue-specific lncRNA and mRNAs detected as outlier expression by applying ROKU [12] to RNA-seq data derived from Human Protein Atlas project [13]. All expression levels were obtained from Expression Atlas (ID: E-MTAB-2836). In total, 6414 lncRNA and 17,806 protein-coding genes with expression level ≥1 FPKM were analyzed in this dataset. The values in parenthesses indicate the ratio of tissue-specific genes to total. (PDF 14 kb)

Additional file 3 Number of tissue-specific lncRNA and mRNAs detected as outlier expression by applying ROKU [12] to RNA-seq data derived from GTEx Consortium [14]. All expression levels were obtained from Expression Atlas (ID: E-MTAB-2919). In total, 6852 lncRNA and 17,612 protein-coding genes with expression level ≥1 FPKM were analyzed in this dataset. The values in parenthesses indicate the ratio of tissue-specific genes to total. (PDF 14 kb)

Additional file 4 Number of tissue-specific lncRNA and mRNAs detected as outlier expression by applying ROKU [12] to RNA-seq data derived from Illumina Body Map project [8]. All expression levels were obtained from Expression Atlas (ID: E-MTAB-513). In total, 5105 lncRNA and 17,017 protein-coding genes with expression level ≥1 FPKM were analyzed in this dataset. The values in parenthesses indicate the ratio of tissue-specific genes to total. (PDF 13 kb)

Additional file 5 Number of tissue-specific lncRNA and mRNAs detected as outlier expression by applying ROKU [12] to RNA-seq data derived from NIH Epigenomics Roadmap project [15]. All expression levels were obtained from Expression Atlas (ID: E-MTAB-3871). In total, 4973 lncRNA and 16,164 protein-coding genes with expression level ≥1 FPKM were analyzed in this dataset. The values in parenthesses indicate the ratio of tissue-specific genes to total. (PDF 14 kb)

Additional file 6 Initial and tissue-specific candidate mRNAs with expression levels ≥1 FPKM for the prediction of TINCR-mRNA interactions. Expression levels were derived from RNA-seq data of Human Protein Atlas project (Expression Atlas ID: E-MTAB-2836). One-tailed Fisher’s exact test was applied for comparing initial dataset and tissue-specific dataset. P-values were adjusted for multiple testing with Bonferroni correction. Tissue-specific expression of TINCR was also detected by ROKU [12]. (PDF 15 kb)

Additional file 7 Our predictions of TINCR-mRNA interactions using 31 different tissue-specific candidate mRNAs. For each tissue, the tissue-specific candidate mRNAs were selected by using RNA-seq data derived from Human Protein Atlas project (Expression Atlas ID: E-MTAB-2836). Combination of two prediction (ranking) methods (MinEnergy and SumEnergy) and two candidate mRNA sets (initial and tissue-specific) were used for the predictions. Experimentally-validated TINCR-mRNA interactions [9] (considered as true positives) were used for evaluating the prediction results. Horizontal axis indicates the number of predicted TINCR-mRNA interactions. Vertical axis indicates the total number of experimentally-validated interactions (true positives). The prediction using skin-specific candidates is already shown in Fig 2. (PDF 51 kb)

Additional file 8 Initial and tissue-specific candidate mRNAs with expression levels ≥1 FPKM for the prediction of TINCR-mRNA interactions. Expression levels were derived from RNA-seq data of GTEx consortium (Expression Atlas ID: E-MTAB-2919). One-tailed Fisher’s exact test was applied for comparing initial dataset and tissue-specific dataset. *P*-values were adjusted for multiple testing with Bonferroni correction. Tissue-specific expression of TINCR was also detected by ROKU [12]. (PDF 19 kb)

Additional file 9 Our predictions of TINCR-mRNA interactions using 30 different tissue-specific candidate mRNAs. For each tissue, the tissue-specific candidate mRNAs were selected by using RNA-seq data derived from GTEx consortium (Expression Atlas ID: E-MTAB-2919). Combination of two prediction (ranking) methods (MinEnergy and SumEnergy) and two candidate mRNA sets (initial and tissue-specific) were used for the predictions. Experimentally-validated TINCR-mRNA interactions [9] (considered as true positives) were used for evaluating the prediction results. Horizontal axis indicates the number of predicted TINCR-mRNA interactions. Vertical axis indicates the total number of experimentally-validated interactions (true positives). (PDF 58.5 kb)

Additional file 10 Summary of RNA-seq data obtained from the human baseline expression data from the Expression Atlas. (PDF 22 kb)

## Abbreviations

FPKM: Fragments per kilobase of exon per million mapped fragments
RIA-Seq: RNA interactome analysis involving deep sequencing
lncRNA: Long non-coding RNA
TINCR: Terminal differentiation-induced ncRNA
