# Supplementary figures and images for: Computational prediction of lncRNA-mRNA interactionsby integrating tissue specificity in human transcriptome
Source: Biol Direct. 2017 Jun 8;12:15. doi: 10.1186/s13062-017-0183-4 (PMC5465533; doi:10.1186/s13062-017-0183-4)

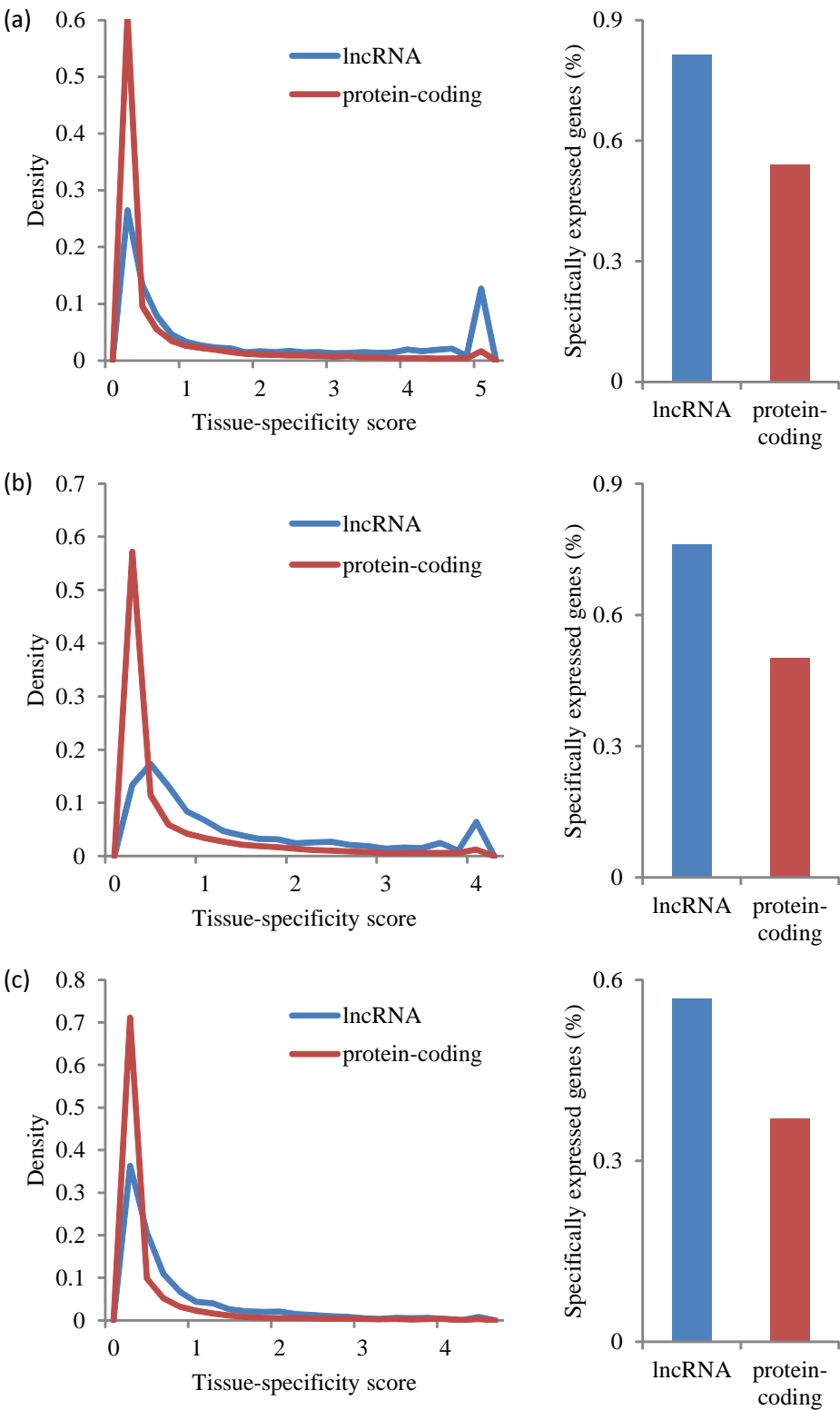

Supplement: Supplementary file 1 — (a) Tissue specificity of 6852 lncRNAs and 17,612 protein-coding genes analyzed using human RNA-seq data from the GTEx Consortium (Expression Atlas ID: E-MTAB-2919). (b) Tissue specificity of 5105 lncRNAs and 17,017 protein-coding genes analyzed using human RNA-seq data from the Human Body Map Project (Expression Atlas ID: E-MTAB-513). (c) Tissue specificity of 4973 lncRNAs and 16,164 protein-coding genes analyzed using human RNA-seq data from the NIH Epigenomics Roadmap project (Expression Atlas ID: E-MTAB-3871). (left) Distributions of tissue-specificity scores [11] calculated for lncRNA and protein-coding genes. (right) Fraction of specifically expressed genes in one or more tissues that were determined to be outliers by ROKU [12]. (PDF 15 kb) [file 13062_2017_183_MOESM1_ESM.pdf]

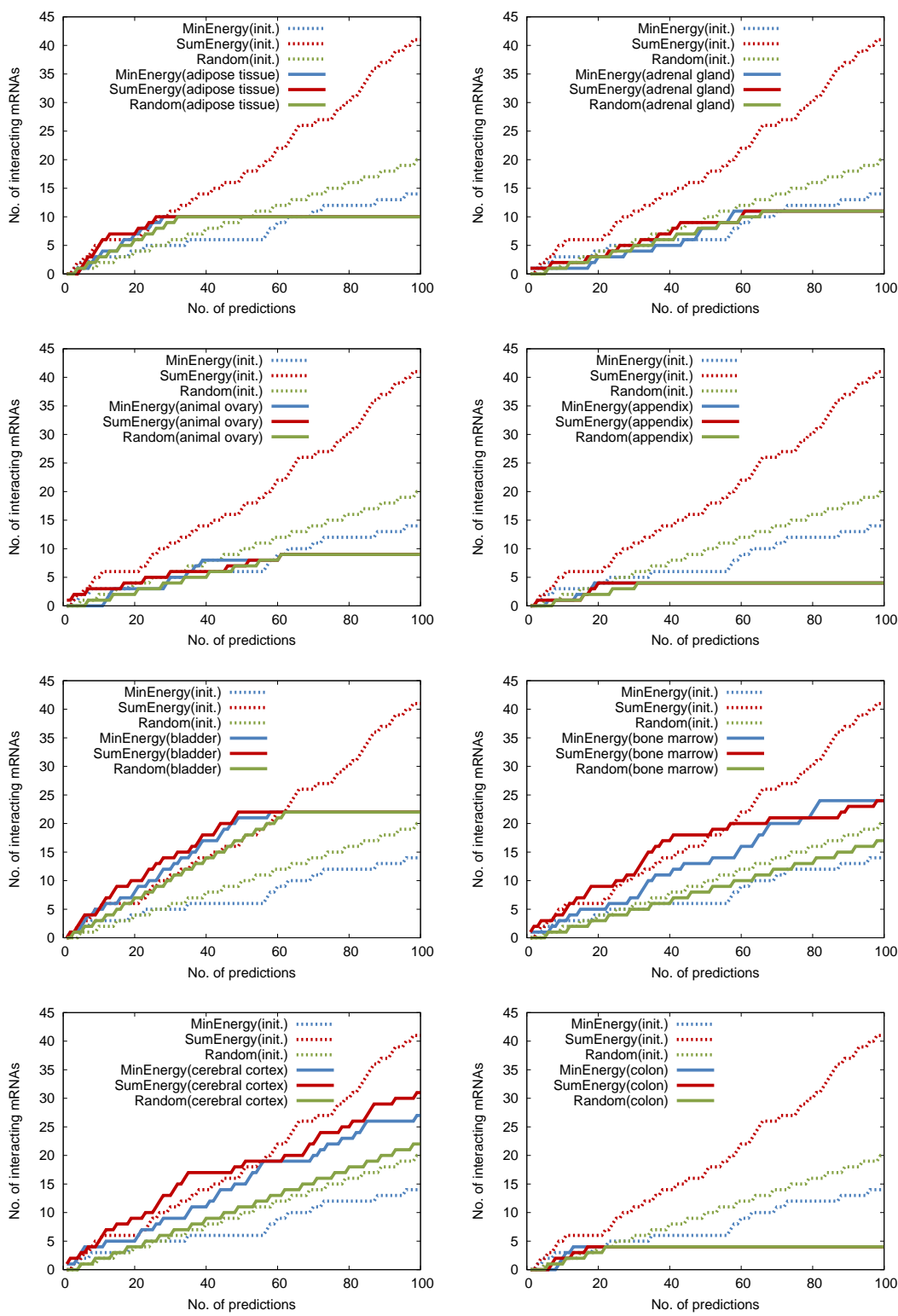

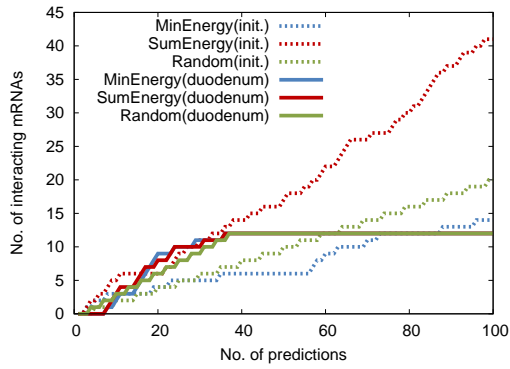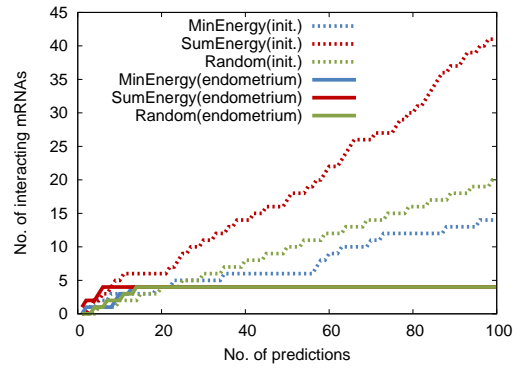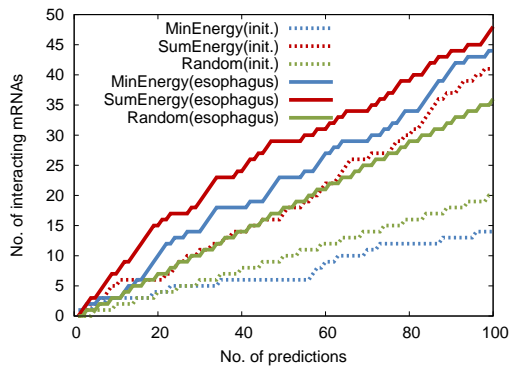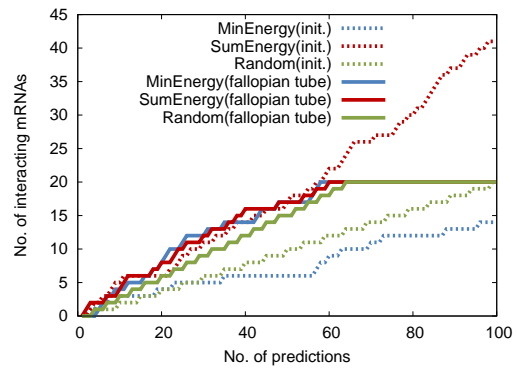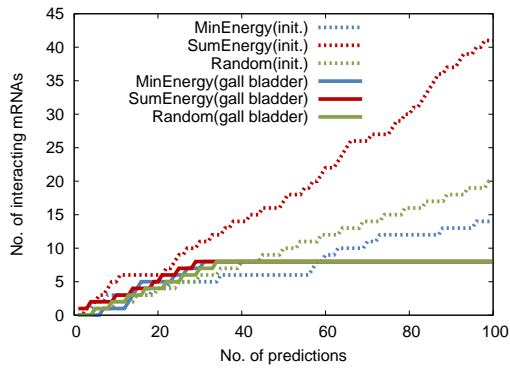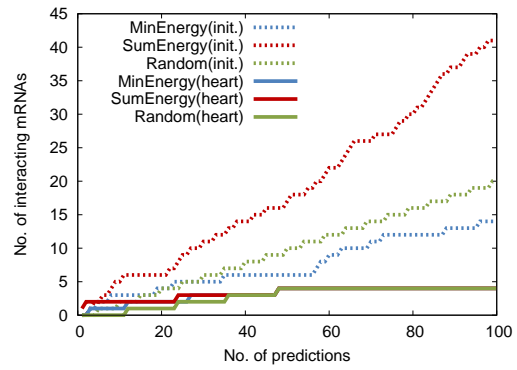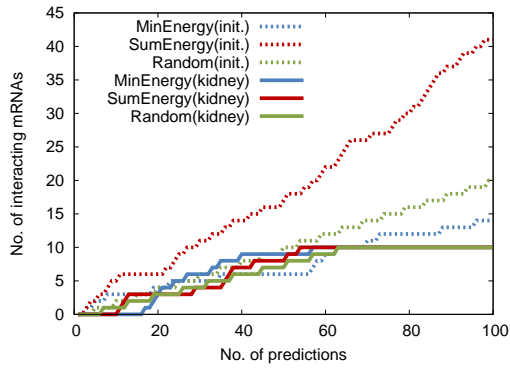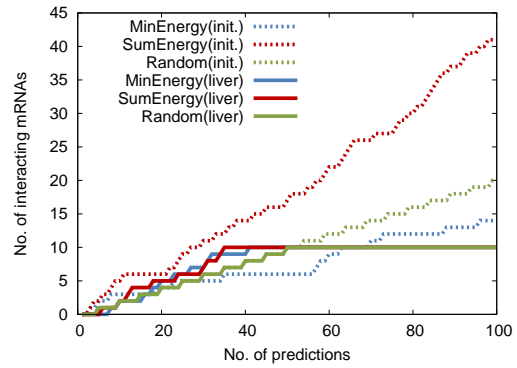

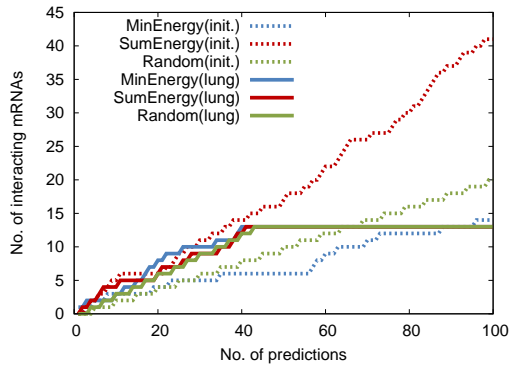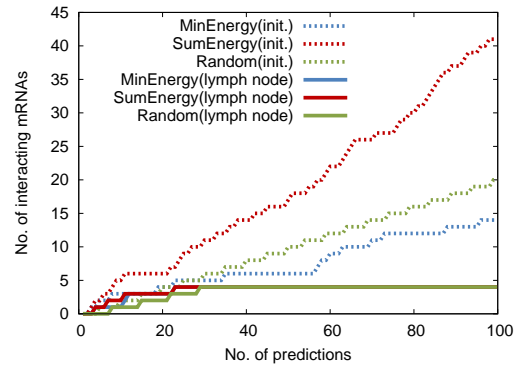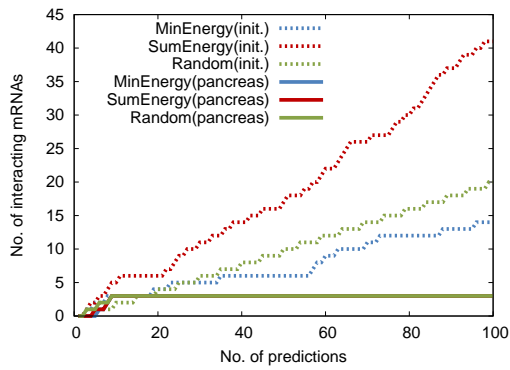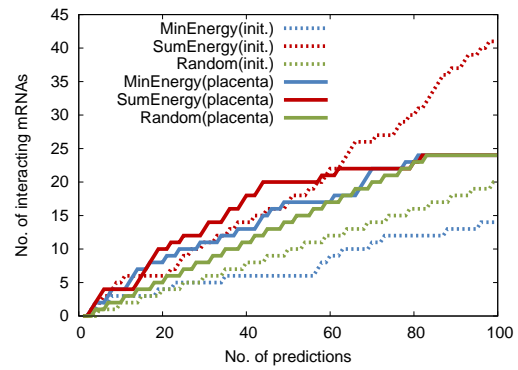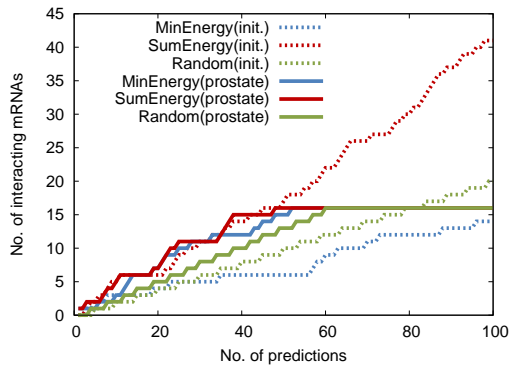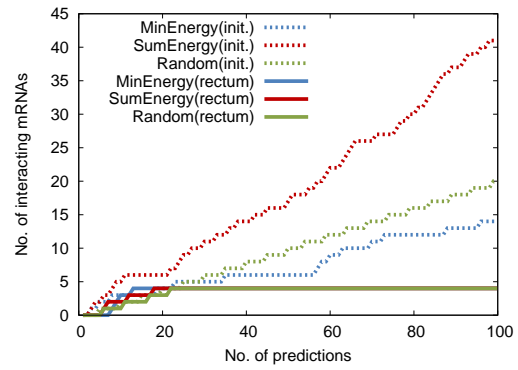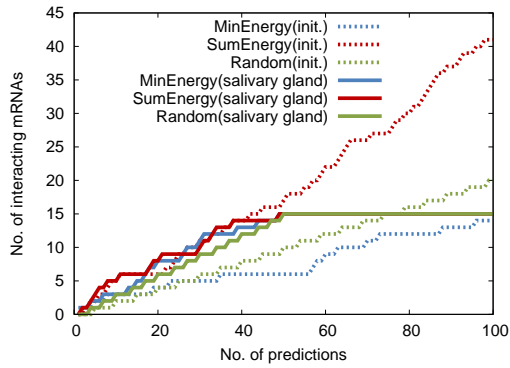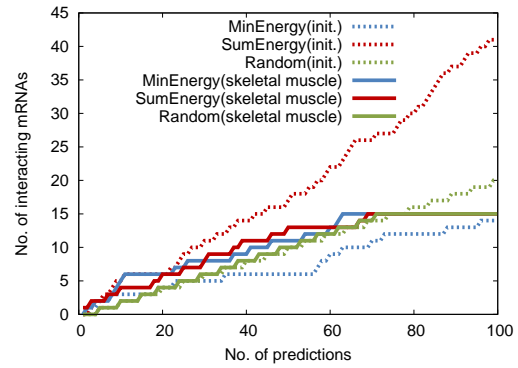

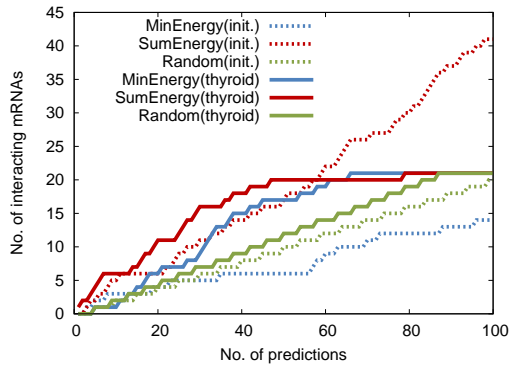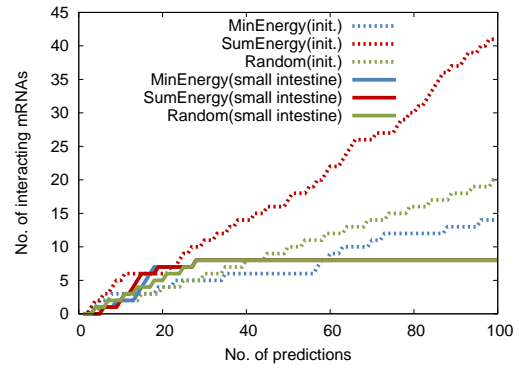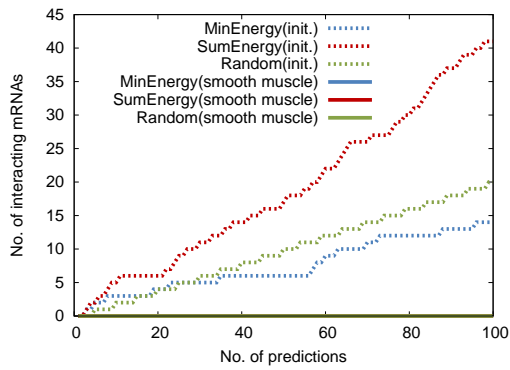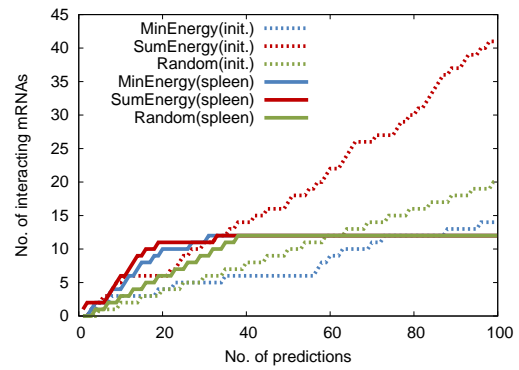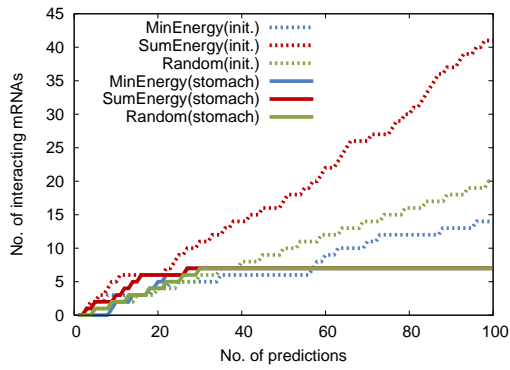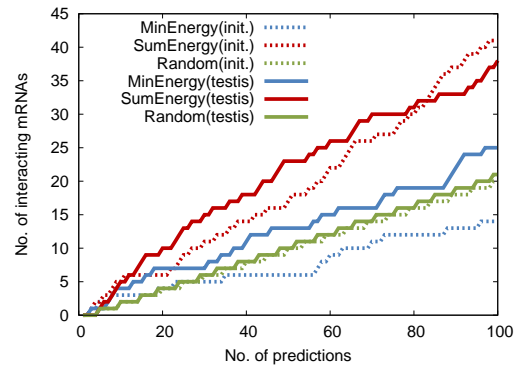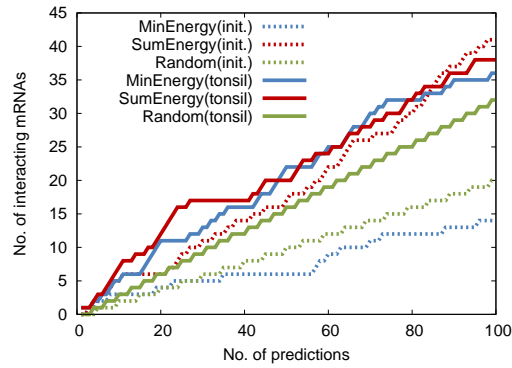

Supplement: Supplementary file 7 — Our predictions of TINCR-mRNA interactions using 31 different tissue-specific candidate mRNAs. For each tissue, the tissue-specific candidate mRNAs were selected by using RNA-seq data derived from Human Protein Atlas project (Expression Atlas ID: E-MTAB-2836). Combination of two prediction (ranking) methods (MinEnergy and SumEnergy) and two candidate mRNA sets (initial and tissue-specific) were used for the predictions. Experimentally-validated TINCR-mRNA interactions [9] (considered as true positives) were used for evaluating the prediction results. Horizontal axis indicates the number of predicted TINCR-mRNA interactions. Vertical axis indicates the total number of experimentally-validated interactions (true positives). The prediction using skin-specific candidates is already shown in Fig 2. (PDF 51 kb) [file 13062_2017_183_MOESM7_ESM.pdf]

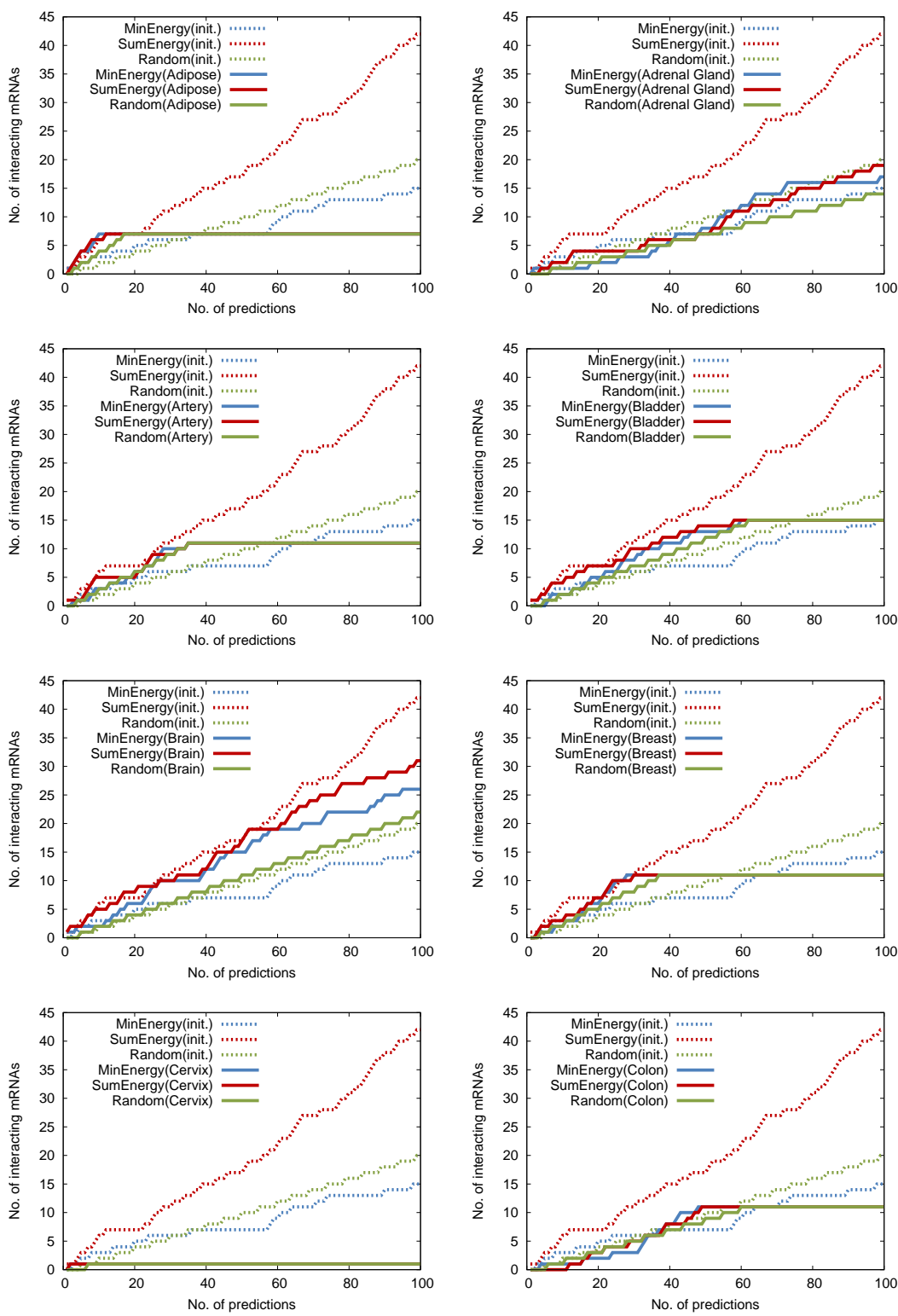

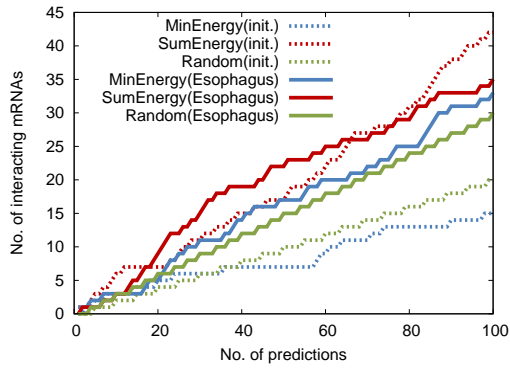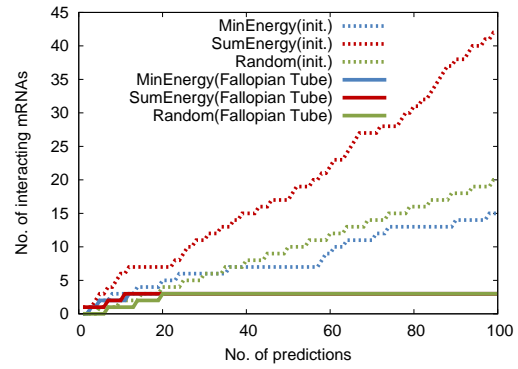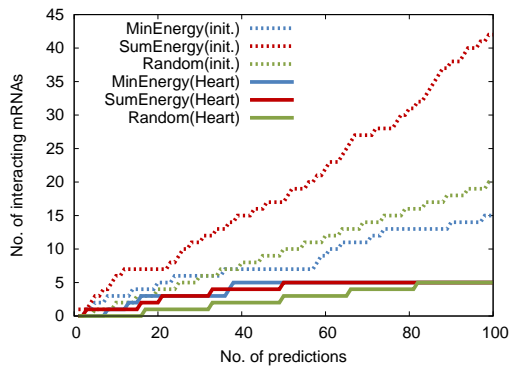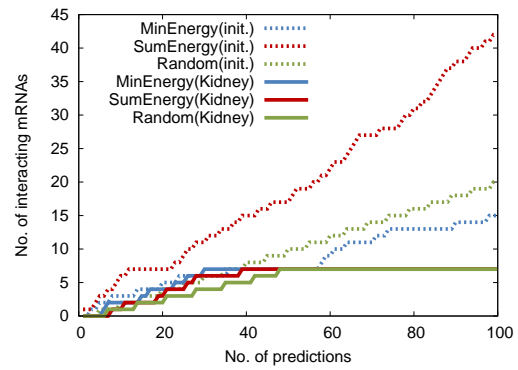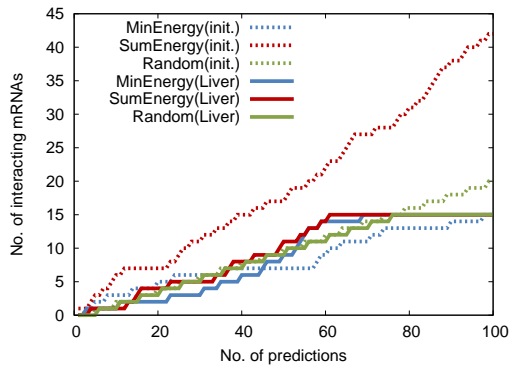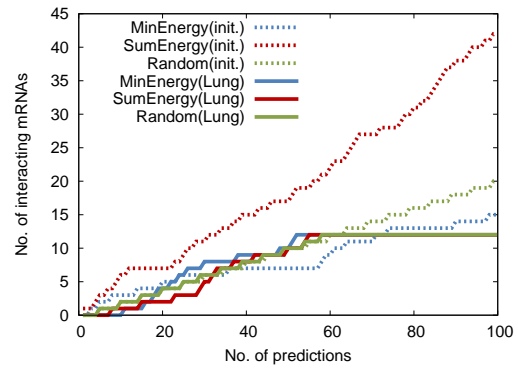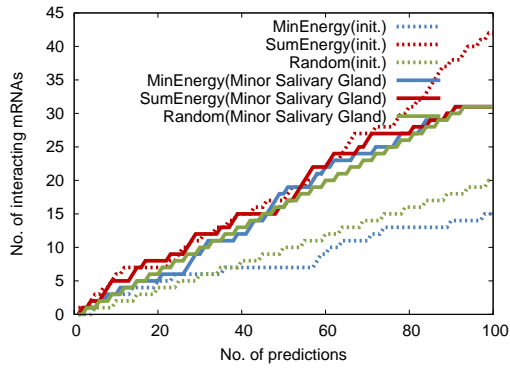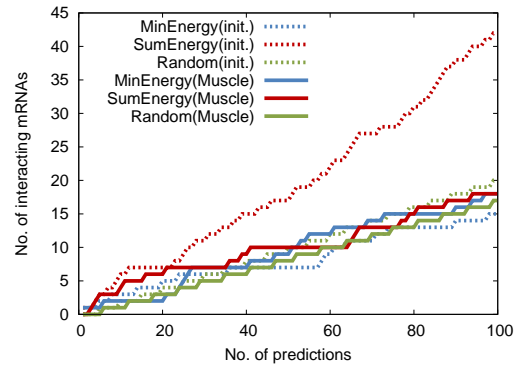

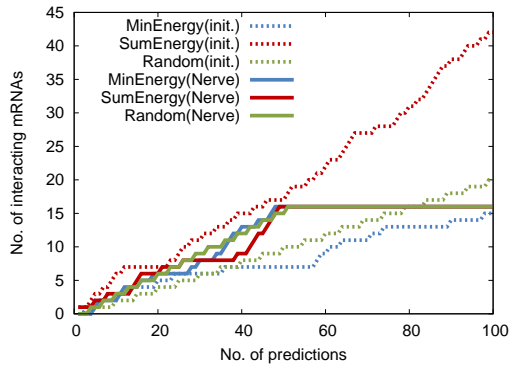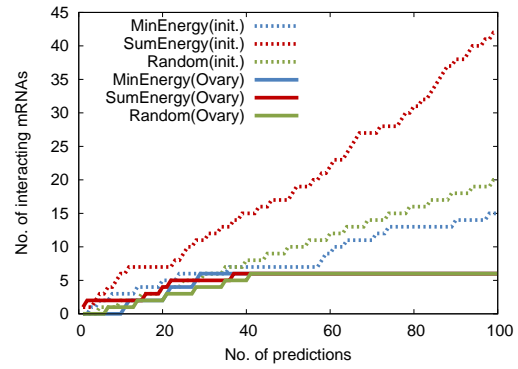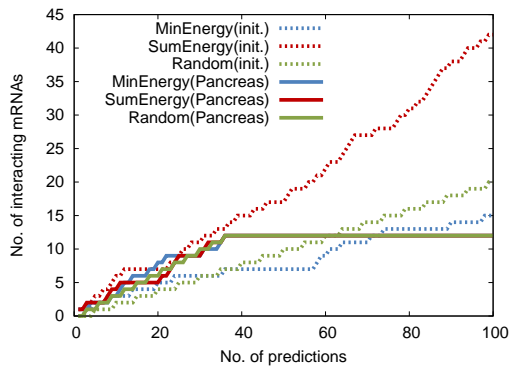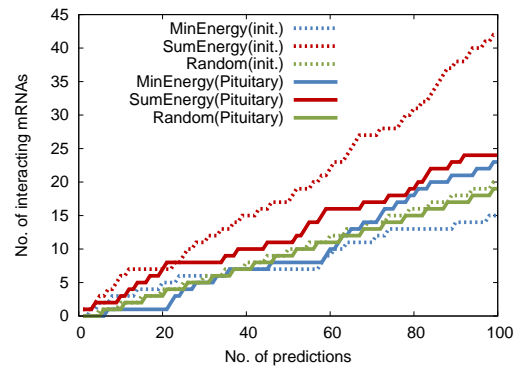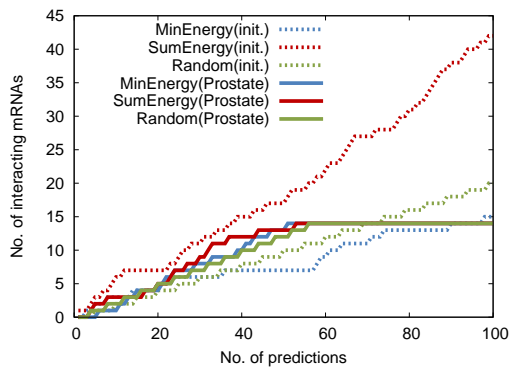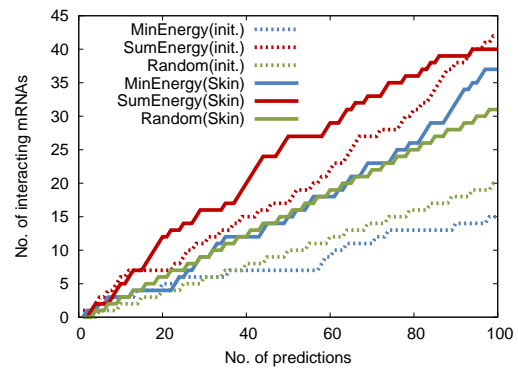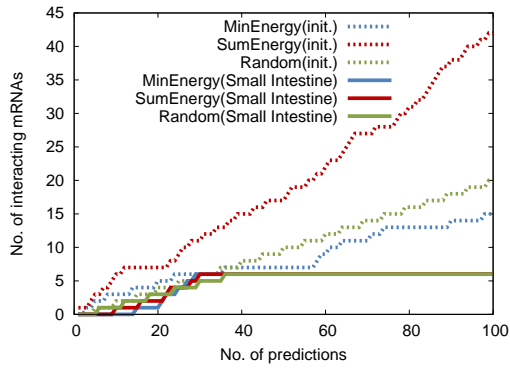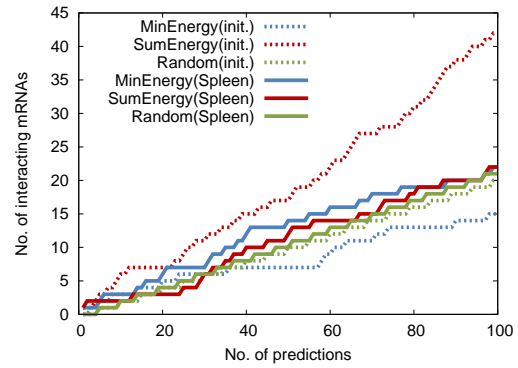

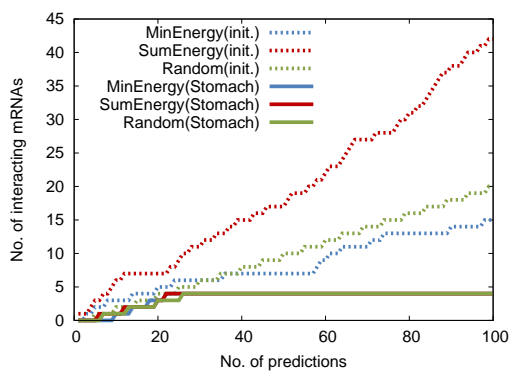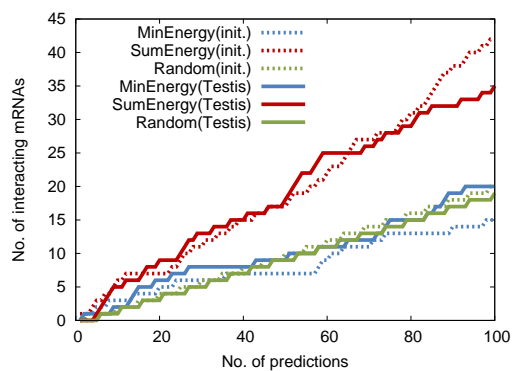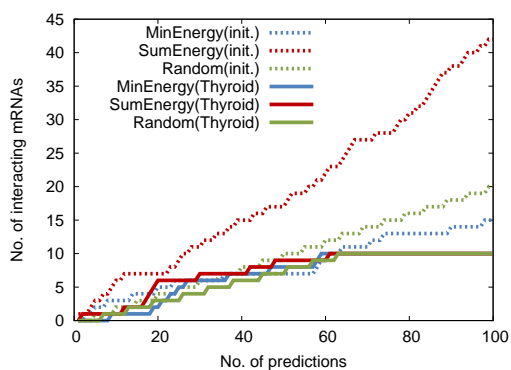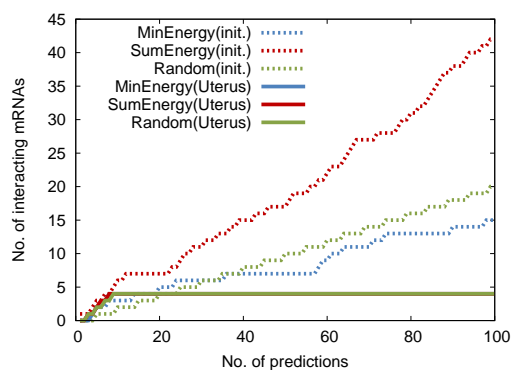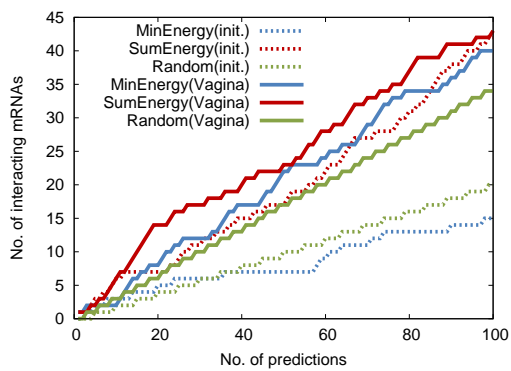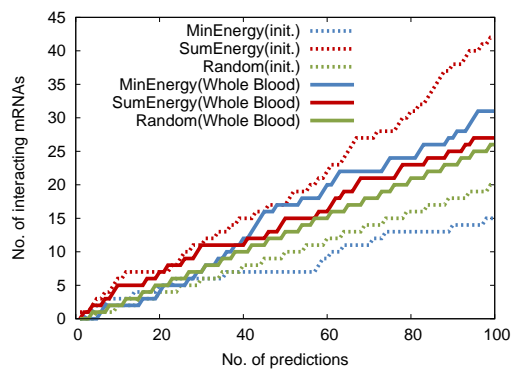

Supplement: Supplementary file 9 — Our predictions of TINCR-mRNA interactions using 30 different tissue-specific candidate mRNAs. For each tissue, the tissue-specific candidate mRNAs were selected by using RNA-seq data derived from GTEx consortium (Expression Atlas ID: E-MTAB-2919). Combination of two prediction (ranking) methods (MinEnergy and SumEnergy) and two candidate mRNA sets (initial and tissue-specific) were used for the predictions. Experimentally-validated TINCR-mRNA interactions [9] (considered as true positives) were used for evaluating the prediction results. Horizontal axis indicates the number of predicted TINCR-mRNA interactions. Vertical axis indicates the total number of experimentally-validated interactions (true positives). (PDF 58.5 kb) [file 13062_2017_183_MOESM9_ESM.pdf]
