# Supplementary material for: Computational prediction of lncRNA-mRNA interactionsby integrating tissue specificity in human transcriptome
Source: Biol Direct. 2017 Jun 8;12:15. doi: 10.1186/s13062-017-0183-4 (PMC5465533; doi:10.1186/s13062-017-0183-4)
Supplement: Supplementary file 2 — Number of tissue-specific lncRNA and mRNAs detected as outlier expression by applying ROKU [12] to RNA-seq data derived from Human Protein Atlas project [13]. All expression levels were obtained from Expression Atlas (ID: E-MTAB-2836). In total, 6414 lncRNA and 17,806 protein-coding genes with expression level ≥1 FPKM were analyzed in this dataset. The values in parenthesses indicate the ratio of tissue-specific genes to total. (PDF 14 kb) [file 13062_2017_183_MOESM2_ESM.pdf]

Additional file 2 — Supplementary Table S1

tissue	lncRNA		mRNA (protein-coding)	
adipose tissue	97	(1.5%)	279	(1.6%)
adrenal gland	158	(2.5%)	670	(3.8%)
animal ovary	228	(3.6%)	446	(2.5%)
appendix	193	(3.0%)	575	(3.2%)
bladder	71	(1.1%)	332	(1.9%)
bone marrow	433	(6.8%)	868	(4.9%)
cerebral cortex	335	(5.2%)	1661	(9.3%)
colon	93	(1.4%)	297	(1.7%)
duodenum	176	(2.7%)	617	(3.5%)
endometrium	150	(2.3%)	249	(1.4%)
esophagus	84	(1.3%)	412	(2.3%)
fallopian tube	294	(4.6%)	735	(4.1%)
gall bladder	90	(1.4%)	397	(2.2%)
heart	110	(1.7%)	433	(2.4%)
kidney	283	(4.4%)	717	(4.0%)
liver	163	(2.5%)	573	(3.2%)
lung	139	(2.2%)	479	(2.7%)
lymph node	236	(3.7%)	493	(2.8%)
pancreas	50	(0.8%)	197	(1.1%)
placenta	180	(2.8%)	529	(3.0%)
prostate	244	(3.8%)	441	(2.5%)
rectum	149	(2.3%)	336	(1.9%)
salivary gland	98	(1.5%)	314	(1.8%)
skeletal muscle	143	(2.2%)	505	(2.8%)
skin	439	(6.8%)	697	(3.9%)
small intestine	165	(2.6%)	540	(3.0%)
smooth muscle	47	(0.7%)	146	(0.8%)
spleen	302	(4.7%)	729	(4.1%)
stomach	108	(1.7%)	419	(2.4%)
testis	2597	(40.5%)	3064	(17.2%)
thyroid	240	(3.7%)	473	(2.7%)
tonsil	188	(2.9%)	667	(3.7%)
