# Supplementary material for: Computational prediction of lncRNA-mRNA interactionsby integrating tissue specificity in human transcriptome
Source: Biol Direct. 2017 Jun 8;12:15. doi: 10.1186/s13062-017-0183-4 (PMC5465533; doi:10.1186/s13062-017-0183-4)
Supplement: Supplementary file 3 — Number of tissue-specific lncRNA and mRNAs detected as outlier expression by applying ROKU [12] to RNA-seq data derived from GTEx Consortium [14]. All expression levels were obtained from Expression Atlas (ID: E-MTAB-2919). In total, 6852 lncRNA and 17,612 protein-coding genes with expression level ≥1 FPKM were analyzed in this dataset. The values in parenthesses indicate the ratio of tissue-specific genes to total. (PDF 14 kb) [file 13062_2017_183_MOESM3_ESM.pdf]

Additional file 3 — Supplementary Table S2

tissue	lncRNA		mRNA (protein-coding)	
Adrenal Gland	141	(2.1%)	586	(3.3%)
Breast	58	(0.8%)	179	(1.0%)
Brain	930	(13.6%)	2045	(11.6%)
Kidney	201	(2.9%)	583	(3.3%)
Cervix	54	(0.8%)	89	(0.5%)
Fallopian Tube	102	(1.5%)	211	(1.2%)
Heart	83	(1.2%)	434	(2.5%)
Liver	190	(2.8%)	709	(4.0%)
Lung	124	(1.8%)	659	(3.7%)
Minor Salivary Gland	75	(1.1%)	462	(2.6%)
Esophagus	114	(1.7%)	597	(3.4%)
Ovary	157	(2.3%)	294	(1.7%)
Pancreas	102	(1.5%)	348	(2.0%)
Pituitary	689	(10.1%)	1425	(8.1%)
Prostate	170	(2.5%)	270	(1.5%)
Muscle	102	(1.5%)	601	(3.4%)
Skin	137	(2.0%)	625	(3.5%)
Spleen	378	(5.5%)	1316	(7.5%)
Stomach	56	(0.8%)	357	(2.0%)
Adipose	70	(1.0%)	180	(1.0%)
Small Intestine	119	(1.7%)	787	(4.5%)
Testis	3331	(48.6%)	3969	(22.5%)
Thyroid	281	(4.1%)	463	(2.6%)
Artery	94	(1.4%)	220	(1.2%)
Nerve	181	(2.6%)	479	(2.7%)
Colon	126	(1.8%)	617	(3.5%)
Bladder	93	(1.4%)	241	(1.4%)
Uterus	67	(1.0%)	124	(0.7%)
Vagina	73	(1.1%)	351	(2.0%)
Whole Blood	202	(2.9%)	1029	(5.8%)
