# Supplementary material for: Computational prediction of lncRNA-mRNA interactionsby integrating tissue specificity in human transcriptome
Source: Biol Direct. 2017 Jun 8;12:15. doi: 10.1186/s13062-017-0183-4 (PMC5465533; doi:10.1186/s13062-017-0183-4)
Supplement: Supplementary file 4 — Number of tissue-specific lncRNA and mRNAs detected as outlier expression by applying ROKU [12] to RNA-seq data derived from Illumina Body Map project [8]. All expression levels were obtained from Expression Atlas (ID: E-MTAB-513). In total, 5105 lncRNA and 17,017 protein-coding genes with expression level ≥1 FPKM were analyzed in this dataset. The values in parenthesses indicate the ratio of tissue-specific genes to total. (PDF 13 kb) [file 13062_2017_183_MOESM4_ESM.pdf]

Additional file 4 — Supplementary Table S3

tissue	lncRNA		mRNA (protein-coding)	
adipose	72	(1.4%)	275	(1.6%)
adrenal	865	(16.9%)	1101	(6.5%)
brain	459	(9.0%)	1830	(10.8%)
breast	162	(3.2%)	444	(2.6%)
colon	68	(1.3%)	298	(1.8%)
heart	124	(2.4%)	459	(2.7%)
kidney	235	(4.6%)	811	(4.8%)
leukocyte	193	(3.8%)	1339	(7.9%)
liver	140	(2.7%)	718	(4.2%)
lung	182	(3.6%)	1085	(6.4%)
lymph node	421	(8.2%)	1005	(5.9%)
ovary	275	(5.4%)	754	(4.4%)
prostate	193	(3.8%)	487	(2.9%)
skeletal muscle	70	(1.4%)	621	(3.6%)
testis	1516	(29.7%)	2695	(15.8%)
thyroid	369	(7.2%)	722	(4.2%)
