# Supplementary material for: Computational prediction of lncRNA-mRNA interactionsby integrating tissue specificity in human transcriptome
Source: Biol Direct. 2017 Jun 8;12:15. doi: 10.1186/s13062-017-0183-4 (PMC5465533; doi:10.1186/s13062-017-0183-4)
Supplement: Supplementary file 5 — Number of tissue-specific lncRNA and mRNAs detected as outlier expression by applying ROKU [12] to RNA-seq data derived from NIH Epigenomics Roadmap project [15]. All expression levels were obtained from Expression Atlas (ID: E-MTAB-3871). In total, 4973 lncRNA and 16,164 protein-coding genes with expression level ≥1 FPKM were analyzed in this dataset. The values in parenthesses indicate the ratio of tissue-specific genes to total. (PDF 14 kb) [file 13062_2017_183_MOESM5_ESM.pdf]

Additional file 5 — Supplementary Table S4

tissue	lncRNA		mRNA (protein-coding)	
adrenal gland	141	(2.8%)	835	(5.2%)
arm muscle	273	(5.5%)	702	(4.3%)
heart	118	(2.4%)	752	(4.7%)
kidney	55	(1.1%)	53	(0.3%)
large intestine	309	(6.2%)	1212	(7.5%)
left kidney	149	(3.0%)	86	(0.5%)
left renal cortex	6	(0.1%)	4	(0.0%)
left renal pelvis	2	(0.0%)	0	(0.0%)
leg muscle	314	(6.3%)	531	(3.3%)
placenta	473	(9.5%)	858	(5.3%)
renal cortex	9	(0.2%)	13	(0.1%)
renal pelvis	28	(0.6%)	23	(0.1%)
right renal cortex	4	(0.1%)	6	(0.0%)
right renal pelvis	2	(0.0%)	3	(0.0%)
small intestine	226	(4.5%)	1072	(6.6%)
spinal cord	492	(9.9%)	1105	(6.8%)
stomach	1049	(21.1%)	1117	(6.9%)
thymus	343	(6.9%)	1717	(10.6%)
trunk muscle	347	(7.0%)	499	(3.1%)
