# Supplementary material for: Computational prediction of lncRNA-mRNA interactionsby integrating tissue specificity in human transcriptome
Source: Biol Direct. 2017 Jun 8;12:15. doi: 10.1186/s13062-017-0183-4 (PMC5465533; doi:10.1186/s13062-017-0183-4)
Supplement: Supplementary file 6 — Initial and tissue-specific candidate mRNAs with expression levels ≥1 FPKM for the prediction of TINCR-mRNA interactions. Expression levels were derived from RNA-seq data of Human Protein Atlas project (Expression Atlas ID: E-MTAB-2836). One-tailed Fisher’s exact test was applied for comparing initial dataset and tissue-specific dataset. P-values were adjusted for multiple testing with Bonferroni correction. Tissue-specific expression of TINCR was also detected by ROKU [12]. (PDF 15 kb) [file 13062_2017_183_MOESM6_ESM.pdf]

Additional file 6 — Supplementary Table S5

Dataset		Interacting	Non-interacting	P-value	TINCR detection
Initial dataset		1034	4056		
Tissue-specific	adipose tissue	10	22	1.0000	
	adrenal gland	11	55	1.0000	
	animal ovary	9	52	1.0000	
	appendix	4	27	1.0000	
	bladder	22	40	0.1421	
	bone marrow	39	185	1.0000	
	cerebral cortex	47	161	1.0000	
	colon	4	18	1.0000	
	duodenum	12	25	1.0000	
	endometrium	4	10	1.0000	
	esophagus	69	121	1.5962E-05	✓
	fallopian tube	20	44	0.8896	
	gall bladder	8	26	1.0000	
	heart	4	44	1.0000	
	kidney	10	53	1.0000	
	liver	10	40	1.0000	
	lung	13	30	1.0000	
	lymph node	4	25	1.0000	
	pancreas	3	6	1.0000	
	placenta	24	59	1.0000	✓
	prostate	16	44	1.0000	
	rectum	4	18	1.0000	
	salivary gland	15	35	1.0000	
	skeletal muscle	15	56	1.0000	
	skin	86	199	0.0031	✓
	small intestine	8	20	1.0000	
	smooth muscle	0	4	1.0000	
	spleen	12	26	1.0000	
	stomach	7	23	1.0000	
	testis	99	367	1.0000	
	thyroid	21	66	1.0000	
	tonsil	50	104	0.0118	
