# Supplementary material for: Computational prediction of lncRNA-mRNA interactionsby integrating tissue specificity in human transcriptome
Source: Biol Direct. 2017 Jun 8;12:15. doi: 10.1186/s13062-017-0183-4 (PMC5465533; doi:10.1186/s13062-017-0183-4)
Supplement: Supplementary file 8 — Initial and tissue-specific candidate mRNAs with expression levels ≥1 FPKM for the prediction of TINCR-mRNA interactions. Expression levels were derived from RNA-seq data of GTEx consortium (Expression Atlas ID: E-MTAB-2919). One-tailed Fisher’s exact test was applied for comparing initial dataset and tissue-specific dataset. P-values were adjusted for multiple testing with Bonferroni correction. Tissue-specific expression of TINCR was also detected by ROKU [12]. (PDF 19 kb) [file 13062_2017_183_MOESM8_ESM.pdf]

Additional file 8 — Supplementary Table S6

Dataset		Interacting	Non-interacting	P-value	TINCR detection
Initial dataset		1034	4056		
Tissue-specific	Adrenal Gland	22	127	1.0000	
	Breast	11	26	1.0000	
	Brain	72	252	1.0000	
	Kidney	7	41	1.0000	
	Cervix	1	6	1.0000	
	Fallopian Tube	3	17	1.0000	
	Heart	5	77	1.0000	
	Liver	15	61	1.0000	
	Lung	12	46	1.0000	
	Minor Salivary Gland	31	62	0.0756	
	Esophagus	81	189	0.0048	✓
	Ovary	6	35	1.0000	
	Pancreas	12	24	1.0000	
	Pituitary	34	142	1.0000	
	Prostate	14	41	1.0000	
	Muscle	30	143	1.0000	
	Skin	79	169	0.0006	✓
	Spleen	23	83	1.0000	
	Stomach	4	22	1.0000	
	Adipose	7	10	1.0000	
	Small Intestine	6	30	1.0000	
	Testis	125	527	1.0000	
	Thyroid	10	53	1.0000	
	Artery	11	24	1.0000	
	Nerve	16	35	1.0000	
	Colon	11	49	1.0000	
	Bladder	15	47	1.0000	
	Uterus	4	5	1.0000	
	Vagina	55	103	0.0007	✓
	Whole Blood	40	112	1.0000	
