# Supplementary material for: Computational prediction of lncRNA-mRNA interactionsby integrating tissue specificity in human transcriptome
Source: Biol Direct. 2017 Jun 8;12:15. doi: 10.1186/s13062-017-0183-4 (PMC5465533; doi:10.1186/s13062-017-0183-4)
Supplement: Supplementary file 10 — Summary of RNA-seq data obtained from the human baseline expression data from the Expression Atlas. (PDF 22 kb) [file 13062_2017_183_MOESM10_ESM.pdf]

Additional file 10 — Supplementary Table S7

Expression Atlas ID	Project	No. of tissues	Skin	Protein-coding[a]	lncRNA[b]	Developmental stage
E-MTAB-2836	Human Protein Atlas	32	Yes	17,806	6,414	adult
E-MTAB-2919[c]	GTEx Consortium	30	Yes	17,612	6,852	adult
E-MTAB-513	Illumina Body Map	16	No	17,017	5,105	adult
E-MTAB-3871	Epigenomics Roadmap	19	No	16,164	4,973	fetuses with congenital defects

[a] The number of protein-coding genes (annotated in GENCODE Release 19) with an expression level >= 1 FPKM in at least one tissue.
[b] The number of lncRNA genes (annotated in GENCODE Release 19) with an expression level >= 1 FPKM in at least one tissue.
[c] This data was derived from several subregions of a tissue. Among these subregions, a representative tissue was arbitrarily selected to reduce this redundancy. In addition, this data also contains two cell line samples. These two samples were also excluded from our analysis.
